# From Pansinusitis to Cerebritis Due to Eikenella corrodens

**DOI:** 10.7759/cureus.54864

**Published:** 2024-02-25

**Authors:** Adriana América Silva, Inês Zão, João A Louro, Eduarda Pereira, Elisabete Monteiro

**Affiliations:** 1 Intensive Care Medicine, Centro Hospitalar do Tâmega e Sousa, Penafiel, PRT; 2 Intensive Care Medicine, Hospital Prof. Doutor Fernando Fonseca, Amadora, PRT; 3 Neurocritical Care, Intensive Care Medicine, Hospital São João, Porto, PRT; 4 Intensive Care Medicine, Centro Hospitalar Universitário de São João, Porto, PRT

**Keywords:** multimodal neuromonitoring, eikenella corrodens, cerebritis, subdural empyema, meningitis

## Abstract

Meningitis is a rare but possible complication of sinusitis. We present a case of a 21-year-old woman with a history of fever, headache and nasal obstruction who presented at the emergency department with psychomotor agitation. Orotracheal intubation and invasive mechanical ventilation were given to protect airway. Blood analysis showed leukocytosis and elevated C-reactive protein. Cerebral and maxillofacial computed tomography (CT) demonstrated pansinusitis with gas foci more prominent in the left frontal sinus with an area of ​​bone rarefaction on the posterior wall with possible communication with the cranial cavity. Lumbar puncture was performed. Empirical antibiotic and corticosteroid therapy were started. Neurosurgery (NC) and Ear Nose and Throat (ENT) surgeons declined indication for urgent surgery and she was admitted at General ICU. On the fourth day of hospitalization, a brain magnetic resonance imaging (CE-MRI) was performed, revealing subdural empyema and cerebritis adjacent to the frontal sinus. She was transferred to the reference neurosurgical center for surgical interventions and was admitted post-operatively at the Neurocritical Care Unit (NCCU). Reevaluation MRI showed residual anterior frontal empyema and absence of focus control in peri-nasal sinusitis, requiring a new ENT surgery. A *Streptococcus *spp was isolated from the blood, *Eikenella corrodens *from the pus collected from the sinuses, and the CSF was sterile. The patient completed 21 days of antibiotic therapy. She was extubated on the 19th day, with Broca's aphasia and right hemiparesis, and on the 23rd day transferred to the ENT Service and later to the Rehabilitation Service.

We present a case of atypical central nervous system (CNS) infection by a rare agent, highlighting the importance of vigilance, focus control, and neurocritical care. In a severe and complex manifestation like this, the management typically involves medical and surgical interventions. Subdural empyema should be treated as a neurosurgical emergency due to the potential rapid deterioration in patient's neurological condition, attributed to secondary damage. In this case, brain multimodal monitoring, was very helpful in acute phase management. Neurocritical care teams should be involved early in patients with this presentation of CNS infection to provide optimal management, reducing complications and secondary brain lesions therefore improving patient outcomes.

## Introduction

Bacterial meningitis and viral encephalitis are severe infections that affect the central nervous system (CNS) with life-threatening risks. In adults, community-acquired bacterial meningitis carries a mortality rate of approximately 20% to 25%, and survivors may be left with a variety of comorbidities [1].

Bacterial meningitis frequently leads to complications involving the nervous system and overall health, including cerebral infarctions, hydrocephalus, septic shock, multi-organ failure, and respiratory distress. Subdural empyema, a less common complication, occurs in approximately 2.7% of cases and should be considered in cases of meningitis occurring alongside otitis or sinusitis [2]. Notably, up to 40 to 80% of patients with subdural empyema also have otolaryngologic infections, particularly in the paranasal sinuses [3]. Complications arising from acute or chronic sinusitis can occur when the infection spreads beyond the sinus cavities. Intracranial subdural empyema primarily affects children and young adults, with a higher incidence in males, where the male-to-female ratio is 3:1 [4,5].

Clinical symptoms of subdural empyema include elevated intracranial pressure and neurological manifestations. Refractory intracranial hypertension with brain herniation and brainstem compression is the most common cause of death [6]. Increased intracranial pressure in bacterial meningitis patients is typically multifactorial, with cytotoxic and interstitial edema due to enhanced permeability of the blood-brain barrier being the primary factor contributing to this condition [7]. High intracranial pressure is indicative of a more severe form of the disease, leading to considerably higher mortality rates compared to uncomplicated cases.

Given the significant morbidity and mortality associated with these conditions, many patients require admission to Intensive Care Units (ICUs). Neurocritical care is focused not only on optimizing treatment for the primary CNS condition but also on mitigating secondary cerebral injuries resulting from factors such as elevated intracranial pressure, seizures, and ischemia.

In this report, we present a case of subdural empyema successfully treated with a combination of medical and surgical interventions at Centro Hospitalar de Universitário São João.

## Case presentation

A 21-year-old woman, with a history of fever for the last 24 hours, headache, and nasal obstruction in the previous week, presented at the emergency department with psychomotor agitation, without focal neurological deficits, with repetitive and incoherent speech (Glasgow Coma Scale (GCS) 13 - eye-opening 4; verbal response 4; motor response 5). Orotracheal intubation and invasive mechanical ventilation were given to protect the airway.

Blood analysis showed leukocytosis (20,000/uL [4500-11000/uL]) and elevated C-reactive protein (150 mg/L [<5.0 mg/L]). She was negative for alcohol, toxins, and illicit drugs.

**Figure 1:**
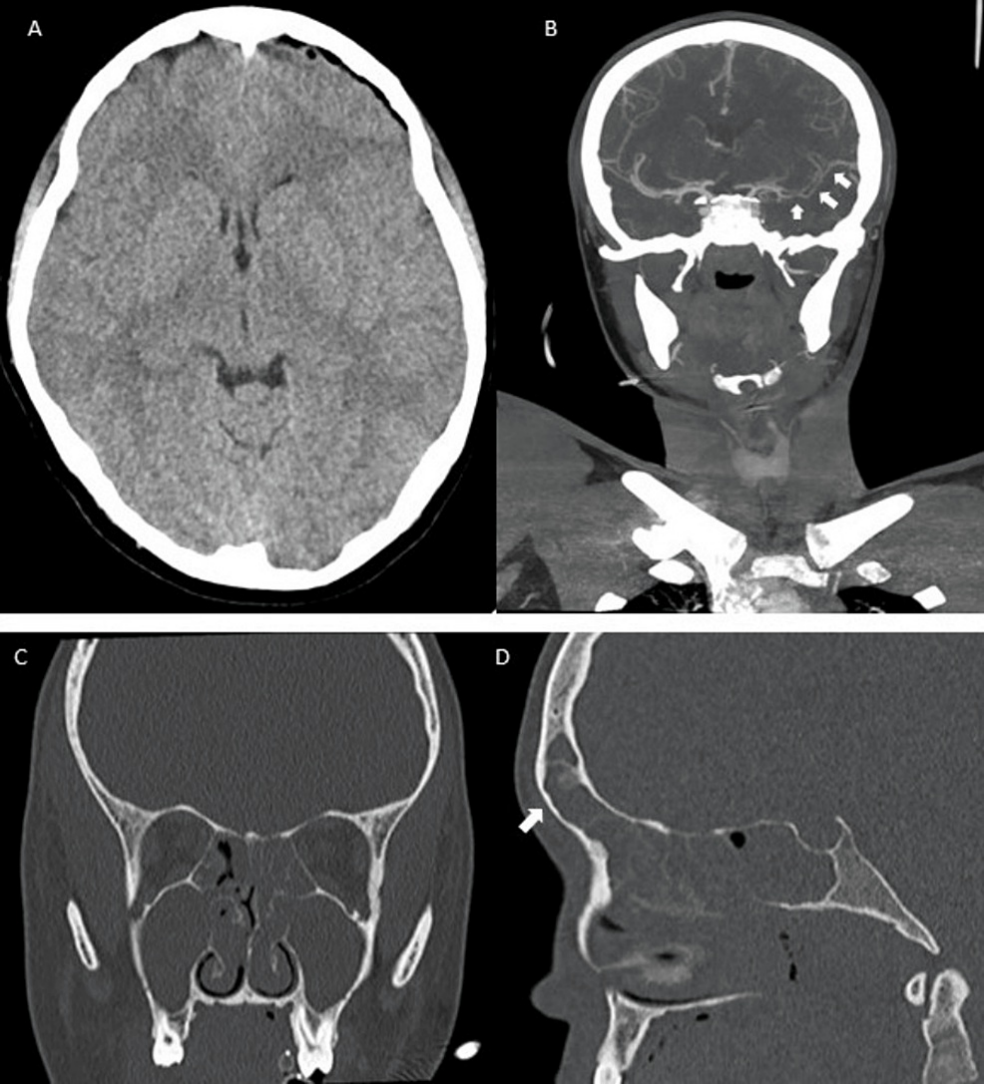
Cerebral computed tomography (CE-CT) angiography and maxillofacial CT at presentation. A - CE-CT imaging. The arrow indicates pneumocephalus; B - Tomography angiography. The arrow indicates reduction in middle cerebral artery segments caliber (M1, M2 and M3); C - Coronal plane maxillofacial CT demonstrates pansinusite; D – Sagittal plane maxillofacial CT. The arrow indicates the left frontal sinusitis.

Cerebral computed tomography (CE-CT) demonstrated inflammatory pansinusitis with gas foci in the left frontal sinus and left frontal extra-axial sinuses; Computed Tomography angiography showed reduction in M1, M2, and M3 (middle cerebral artery segments) caliber in a probable context of vasculitis. Maxillofacial CT confirmed an extensive inflammatory component of the paranasal sinuses bilaterally, with gas foci more prominent in the left frontal sinus with an area of ​​bone rarefaction on the posterior wall with possible communication with the cranial cavity (Figure 1).

A lumbar puncture was performed, and a cloudy cerebrospinal fluid (CSF) was obtained: 132 cells/microliter (91% neutrophils), proteins of 222 mg/dl, and glucose of 77 mg/dl (57% of serum glucose). CSF was collected for microbiology and for polymerase chain reaction (PCR) for* Pneumococcus *spp, *Meningococcus *spp, *Haemophilus *spp, parvovirus, enterovirus, adenovirus, herpes simplex viruses 1, 2 and varicella-zoster virus. Blood cultures were done. Empirical antibiotic therapy was started with ceftriaxone 2 grams each 12 hours (h), metronidazole 500 milligrams (mg) every six hours, and corticosteroid therapy with dexamethasone 6 mg/day.

Neurosurgery (NC) and Ear Nose and Throat (ENT) surgeons declined indication for urgent surgery and she was admitted to the General ICU. She was sedated for Richmond Agitation Sedation Scale (RASS) -4/-5, apyretic, with a sustained decrease in inflammatory markers, with no organic dysfunctions.

**Figure 2:**
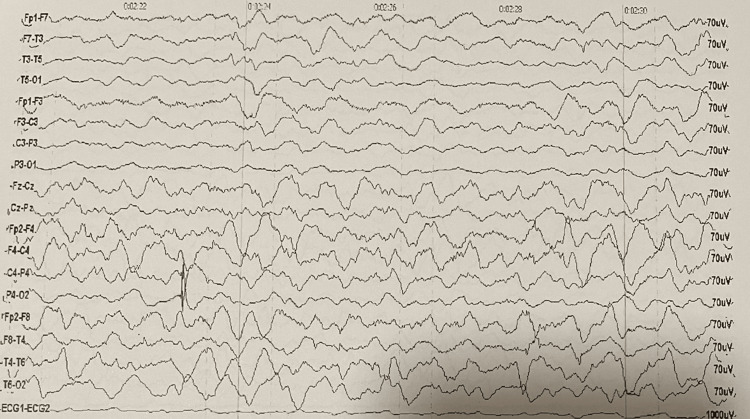
EEG at day 1. An overall very slow (delta 0.5-3 Hz) and broad (130-150 mV) pattern, with attenuation of voltage in the left hemisphere, translating diffuse cerebral dysfunction with maximum expression in the left hemisphere.

Electroencephalogram (EEG) on the first day showed diffuse cerebral dysfunction, mainly in the left hemisphere, without epileptic activity (Figure 2).

**Figure 3:**
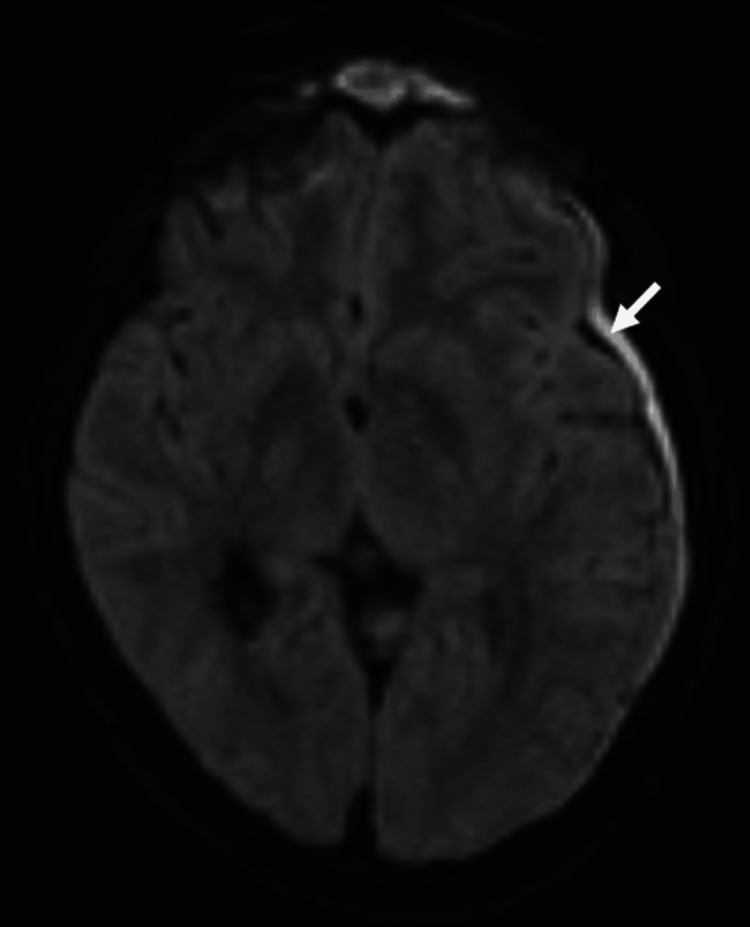
Diffusion-weighted (DWI) magnetic resonance imaging. The arrow indicates the subdural empyema.

On the fourth day of hospitalization, brain magnetic resonance imaging (CE-MRI) was performed, revealing subdural empyema and cerebritis adjacent to the frontal sinus (Figure 3). She was referred again to Neurosurgery and she was transferred to the reference neurosurgical center for neurosurgical and ENT interventions.

She was submitted to craniotomy for left hemispheric subdural empyema drainage, trepanation of the left frontal sinus, septoplasty and bilateral sinonasal endoscopic surgery. A large amount of purulent exudate from the left frontal and ethmoid sinuses bilaterally was drained. Left frontal drain and intracranial pressure (ICP) sensor were placed, initial ICP 9 mmHg.

She was admitted post-operatively at the Neurocritical Care Unit (NCCU) and multimodal brain monitoring was given: ICP sensor, optimal cerebral perfusion pressure (CPP) according to pressure reactivity index (PRx), and near-infrared spectroscopy (NIRS).

On the second postoperative day, she developed intracranial hypertension, managed with osmotherapy, after that, she maintained normal ICP values, requiring norepinephrine for 12 days to ensure target CPP according to the cerebral autoregulation curve.

On the ninth day of hospitalization, a reevaluation Brain MRI (Figure 1) and peri-nasal sinuses MRI and CT showed residual anterior frontal empyema, leptomeningeal uptake of the left hemispheric sulci with hyper signal of the cortex, and hypo intensity of the subcortical white matter on T2/FLAIR, translating meningitis/cerebritis, deformation of the ventricular system and deviation of the median structures to the right by approximately 9 mm, dilation of the right lateral ventricle due to active hydrocephalus and signs of periventricular interstitial edema and maintenance of an acute infectious process in the maxillary sinus, left sphenoid chamber and frontal sinus. A new ENT surgery was performed to control the infectious focus.

Microbiologically, it was isolated a *Streptococcus *spp in the blood, in the pus collected from the sinuses, and *Eikenella corrodens* (group HACEK) and the CSF was sterile. A transthoracic echocardiogram showed no evidence of endocarditis. The patient completed 21 days of antibiotic therapy with ceftriaxone and metronidazole and seizure prophylaxis with levetiracetam 500 mg every 12 hours.

**Figure 4:**
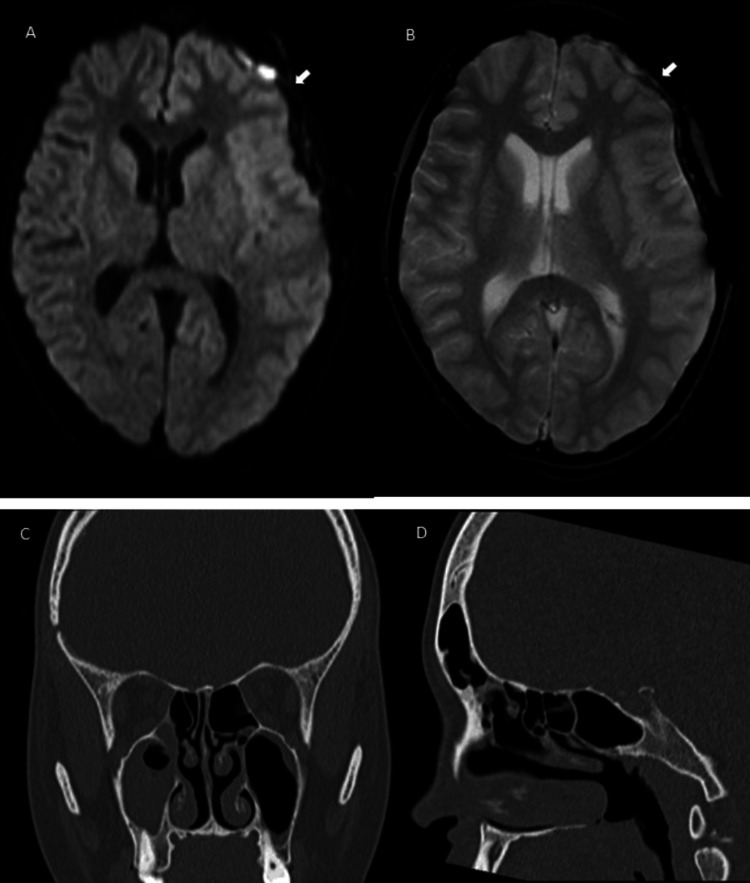
Brain MRI and peri-nasal sinuses CT five days after the last surgery. A - Diffusion-weighted (DWI) magnetic resonance imaging. The arrow indicates residual left frontal subdural empyema; B - Gradient recalled echo (GRE) T2 magnetic resonance imaging. The arrow indicates residual left frontal subdural empyema; C - Coronal plane maxillofacial CT demonstrate decreased filling of the paranasal sinuses; D - Sagittal plane maxillofacial CT. The arrow indicates the left frontal sinus.

Brain MRI and peri-nasal sinuses CT (Figure 4) five days after the last surgery demonstrated decreased filling of the paranasal sinuses, residual left frontal subdural empyema, resolution of the mass effect on the left lateral ventricle, and absence of hydrocephalus.

She was extubated on the 19th day, and recovered consciousness, with Broca's aphasia and right hemiparesis (grade 3 in the upper limb, grade 4 in the lower limb). She was transferred from the NCCU on the 23rd day of hospitalization to the ENT Service and later to the Rehabilitation Service to continue the motor rehabilitation program, speech therapy, occupational therapy, and neuropsychology. Finally, she was discharged from the hospital on the 38th day, with improvement in aphasia and right hemiparesis (grade 4).

## Discussion

In this report, we present a case of atypical CNS infection by a rare agent, highlighting the importance of vigilance, focus control, and neurocritical care.

Meningitis is a rare but possible complication of sinusitis. It is a serious infection that, despite medical advances, still carries a high mortality rate. On the other hand, rhinosinusitis is usually a condition that resolves on its own, but in rare instances, it can lead to life-threatening complications.

Both acute and chronic rhinosinusitis can have viral or bacterial origins. Viral infections are commonly attributed to rhinoviruses or coronaviruses, while bacterial infections often involve *Streptococcus pneumoniae*, *Hemophilus influenzae*, and *Moraxella catarrhalis* [8,9].

Complications arising from rhinosinusitis can be categorized as orbital (60-80%), intracranial (15-20%), or osseous (5%) [8]. Bacterial complications in adults are rare, with an estimated global incidence of three cases per million people annually [10]. Intracranial complications, including meningitis, epidural empyema, subdural empyema, cerebritis, brain abscess, superior sagittal and cavernous sinus thrombosis, can affect individuals of all ages, although young adult males in their second decade are most commonly affected [8]. Infections can spread through two routes. First, via retrograde spread, facilitated by thrombus formation or the release of septic emboli traveling through valve-less diploic veins to reach the brain. Second, a direct extension of the disease may occur through the erosion of thin osseous sinus walls, congenital or traumatic defects, or existing foramina [8].

Subdural empyema is an uncommon infection that occurs within the intracranial space, situated between the dura mater and the arachnoid mater. These infections can lead to the development of mass effects, focal neurological impairments, seizures, coma, and, in severe cases, even fatalities. Importantly, they not only induce mass effects but also instigate inflammatory reactions, potentially leading to conditions like vasospasm, encephalitis, and thrombosis in cortical veins. In a review by Jim et al. [11] subdural empyema was a complication in 2.7% of community-acquired bacterial meningitis, with 75% of patients also having concurrent otitis or sinusitis. *Eikenella corrodens*, a Gram-negative bacillus that is part of the normal flora of the mouth, upper respiratory tract, lower gastrointestinal tract, and female genital tract was rarely associated with CNS, particularly brain abscesses [12,13]. Lasso et al. in another review [14] revealed approximately 34 cases of CNS involvement with abscess formation due to *Eikenella corrodens* worldwide since 1970.

In a severe and complex manifestation like this, the management typically involves medical and surgical interventions such as targeted high-dose intravenous antimicrobial therapy and focus control with drainage of the empyema and infected sinuses. The prompt surgical drainage of affected sinuses is imperative as a focus control strategy. Subdural empyema should be treated as a neurosurgical emergency due to the potential rapid deterioration in the patient's neurological condition, attributed to ischemic and inflammatory changes, thrombosis, and edema beneath the empyema. Neurosurgical intervention not only reduces the volume of pus but also alleviates elevated intracranial pressure and assists in identifying the causative organism, facilitating adjustments to the antibiotic regimen. Early introduction of broad-spectrum antibiotics, adaptable based on antibiogram results, is essential. In bacterial meningitis, inflammation and release of factors that induce direct cellular toxicity and disruption of the blood-brain barrier result in cytotoxic and vasogenic edema, raising ICP [7]. ICP elevation induces secondary brain injury by reducing cerebral perfusion pressure, leading to ischemia.

In this case, ICP and autoregulation monitoring, by using PRx, as a part of brain multimodal monitoring, was key to managing the acute phase. Brain multimodal monitoring offers the evaluation of cerebral vascular autoregulation, and optimal CPP and contributes to achieve adequate cerebral blood flow and oxygenation. No randomized controlled trials have investigated the usefulness of this type of monitoring in comatose patients with CNS infections, but it could be important in this group since it provides continuous and bedside critical information for timely interventions, helps to prevent secondary brain injury, and improves outcomes.

## Conclusions

In CNS infections, early diagnosis and multidisciplinary management are crucial for effective focus control, morbidity and mortality reduction, and prevention of complications. Neurocritical care teams should be involved early to provide optimal management using multimodal neuromonitoring and avoid secondary brain injury, reduce complications and improve patient outcomes.
